# Composite Fault Feature Index-Guided Variational Mode Decomposition with Dynamic Weighted Central Clustering for Bearing Fault Detection

**DOI:** 10.3390/s26041394

**Published:** 2026-02-23

**Authors:** Bangcheng Zhang, Boyu Shen, Zhi Gao, Yubo Shao, Zaixiang Pang, Xiaojing Yin

**Affiliations:** School of Mechatronic Engineering, Changchun University of Technology, Changchun 130103, China; shenboyu1209@163.com (B.S.); gaozhi@ccut.edu.cn (Z.G.); shaoyubo@ccut.edu.cn (Y.S.); pangzaixiang@ccut.edu.cn (Z.P.)

**Keywords:** bearing fault, fault characteristic, modal decomposition, clustering algorithm

## Abstract

To address the periodic impacts and amplitude-modulated high-frequency resonance phenomena caused by bearing faults in rotating machinery, this paper proposes a detection method. The core innovation lies in: firstly, constructing a composite fault feature index (CFFI) that integrates normalized kurtosis and fuzzy entropy, which synchronously quantifies the fault impact intensity and periodic structure, and serves as an optimization objective; secondly, definining a spectral energy retention rate (SERR) that includes both the full spectrum and characteristic frequency bands to evaluate the denoising effect and fault feature retention, respectively. Based on this, the method adaptively determines the Variational Mode Decomposition (VMD) parameters through the Triangular Topology Aggregation Optimizer (TTAO), and uses Dynamic Weighted Center Clustering (DWCC) to screen key IMFs containing fault-envelope information. On the IMS bearing dataset, the SERR of the reconstructed signal is 0.21356, which is higher than the actual collected signal value of 0.22465, with a relative error of 4.9%, indicating a higher reconstruction accuracy. These quantitative results indicate that CFFI-guided optimization enhances impulsive and periodic fault components while maintaining stable feature-band retention. This approach is suitable for real-world equipment monitoring and exhibits strong engineering applicability.

## 1. Introduction

As a critical component of rotating machinery, rolling bearing failure often manifests as periodic impacts between rolling elements and defect sites. This process excites high-frequency resonance, generating impulses and amplitude-modulation structures in the envelope domain [1,2,3]. Under real-world conditions characterized by variable operating states, intense noise, and multi-source interference, fault features are typically weak, non-stationary, and easily masked, posing significant challenges to the sensitivity and robustness of traditional diagnostic methods that rely on fixed parameters and single features [4]. Consequently, how to adaptively select frequency bands, achieve stable signal decomposition, and quantitatively evaluate feature preservation in real-world scenarios has become a key research direction in bearing fault signal processing.

Classical diagnostic procedures typically follow the “band selection–envelope demodulation” principle. Methods such as spectral kurtosis and kurtograms are first used to select resonance bands that highlight transients, followed by zero-phase bandpass filtering and a Hilbert transform to obtain the envelope, thereby enhancing impulsive and modulated fault information [5]. While effective in many engineering applications, this fixed-parameter approach with a single band-selection criterion is prone to band-selection bias and feature degradation under low signal-to-noise ratio (SNR) and non-stationary conditions [6]. To enhance feature separation, adaptive time–frequency decomposition methods have been widely adopted. Techniques such as EMD, EEMD, and CEEMDAN can decompose signals into intrinsic modes at different scales and capture transients, but they suffer from issues such as mode mixing and end effects [7]. In contrast, Variational Mode Decomposition (VMD) is an adaptive signal decomposition method that recursively extracts intrinsic mode functions (IMFs) with specific center frequencies by constructing and solving a constrained variational problem. VMD iteratively solves for modes and their center frequencies within a variational framework that minimizes bandwidth, offering better noise robustness and decomposition stability, which aligns well with the “impact-resonance” signal mechanism [8]. However, the decomposition quality of VMD is susceptible to its penalty factor (α) and mode number (K). The penalty factor (α) controls the bandwidth of each extracted mode; a larger α yields smoother modes with better noise suppression but may obscure impulsive features, while a smaller α preserves detail at the cost of increased noise sensitivity. The mode number (K) determines how many intrinsic mode functions (IMFs) are generated; an insufficient K leads to under-decomposition (merging of distinct fault components), whereas an excessive K causes over-decomposition (splitting of fault features and introduction of spurious components). In bearing fault diagnosis, an inappropriate penalty factor or mode number (α, K) can directly obscure the impulse-induced resonance signatures targeted for extraction. Without data-driven criteria, over-decomposition or under-decomposition can easily occur.

To address VMD’s parameter sensitivity, recent research has employed metaheuristic optimization algorithms, using a health indicator as the fitness function to achieve adaptive parameter tuning. This approach demonstrates a good balance between global and local search in selecting signal-processing parameters [9,10]. Meanwhile, entropy-based features (e.g., sample entropy, permutation entropy, fuzzy entropy) and kurtosis are complementary in characterizing the complexity and impulse strength of time series. They are often used as fault-sensitive indicators or combined into composite criteria to enhance sensitivity and robustness to weak features [11,12]. In the post-decomposition stage, clustering methods can help distinguish noise-dominant from fault-dominant intrinsic mode functions (IMFs), reducing the impact of mode mixing on reconstruction quality and thus preserving fault-related components more effectively. For evaluation, publicly available datasets, such as the IMS bearing data, provide standardized benchmarks and multi-condition coverage for generalized validation [13].

In addition, in terms of constructing health indicators, although there have been studies combining multiple features [14], they are mostly used for later diagnosis and rarely used as a unified optimization criterion for guiding the entire process of signal decomposition [15]. In the stage of modal selection and reconstruction, traditional methods often rely on empirical thresholds or simple rules to screen IMFs, lacking robustness analysis of IMF cluster characteristics, and are susceptible to mode aliasing and noise IMF interference [16].

To tackle the aforementioned challenges, this paper constructs a closed-loop diagnostic framework following the sequence: “indicator, decomposition, clustering, reconstruction, evaluation.” Specifically: (1) Guided by spectral kurtosis, perform frequency band selection, followed by zero-phase filtering and envelope demodulation. (2) Use the proposed Composite Fault Feature Indicator (CFFI), which integrates impulse sensitivity with complexity robustness, as the fitness function to optimize VMD’s α and K parameters adaptively. This focuses the decomposition on components related to fault envelopes. (3) Apply Dynamic Weighted Central Clustering (DWCC) to the obtained IMFs and reconstruct the fault-dominant components. (4) Quantify noise reduction and feature preservation using a defined Spectral Energy Retention Rate (SERR). The framework’s stability and engineering applicability are validated using the IMS bearing dataset. A comparative analysis with baseline methods using various entropy-based fitness functions demonstrates the superior robustness of the proposed approach.

The main contributions of this paper are as follows:

1. CFFI integrating features sensitive to impulses and robust to signal complexity, serving as a unified criterion for both VMD parameter optimization and IMF selection.
2. Employs health-indicator-driven metaheuristic optimization to adaptively select VMD’s α and K, mitigating over-/under-decomposition and enhancing focus on fault-envelope information.
3. Adopts DWCC for robust clustering and reconstruction of IMFs, reducing the effects of mode mixing and highlighting fault-dominant components.
4. Introduces the SERR as an evaluation metric, establishing a closed-loop quantitative assessment from band selection to reconstruction, facilitating online monitoring and trend tracking.

## 2. Research Methodology

The objective of this study is to achieve real-time detection of bearing faults (e.g., spalling on inner/outer rings or rolling elements) by determining optimal VMD parameters. This guides the adaptive selection of the impact and modulated resonance information inherent to such faults [17,18]. This objective is achieved through four key steps: (1) A CFFI specific to bearing faults is proposed to quantitatively measure the strength of fault-related impulses and periodic structures, simultaneously serving as the fitness function for the optimization algorithm. (2) The selected fitness function is used to optimize the two key VMD parameters: the penalty factor α and the mode number K. This optimization ensures that the decomposed IMFs are concentrated within the fault-related resonance bands and envelope components, thereby adaptively amplifying bearing fault signatures. (3) An improved clustering method, DWCC, is applied to the IMFs. Based on the CFFI values, the cluster with the highest average CFFI is selected for signal reconstruction, yielding a signal enriched with bearing fault features. (4) Finally, the feature-band SERR is calculated within the known or estimated fault characteristic frequency band Ω, enabling the identification and trend tracking of bearing fault characteristics.

### 2.1. Health Indicators

In fault diagnosis systems, health indicators are the key to converting raw vibration signals into interpretable, state-sensitive quantitative measures. Their core function is to detect, quantify, and track early fault characteristics, especially in noisy and non-stationary operating conditions, where simple visual or statistical analysis often fails. To effectively capture the key changing characteristics of bearing fault signals, this paper proposes a novel CFFI.

Traditional health indicators, such as root mean square (RMS), KI, and various entropy indicators, are often designed for the single physical characteristics of signals. Although effective in specific scenarios, their sensitivity and robustness are difficult to balance when facing the complex characteristics of impact and periodic modulation that coexist in bearing faults. For example, kurtosis is sensitive to impact but susceptible to noise and outlier interference; The entropy index has a certain ability to characterize periodic structures, but its peak response to transient shocks is weak.

This makes CFFI not only a better-performing alternative indicator, but also a closed-loop guiding core that embeds diagnostic intelligence into the entire process of “decomposition, optimization, clustering, evaluation”. The application of traditional indicators is often open-loop and isolated, while CFFI optimizes and filters signals through the same criteria, improving the cohesion, adaptability, and target consistency of the entire feature extraction process. In addition, in Section 4, this article compares CFFI with the traditional metrics root mean square (RMS) and KI in three evaluation dimensions: trend (Tred), monotonicity (Mono), and scale-adaptive comprehensive feature variability (SAIF).

#### 2.1.1. Construction of the Composite Fault Feature Index

The introduction of the CFFI is based on an understanding of the limitations of existing signal-feature extraction methods, and its construction is crucial to the adaptiveness of the optimization algorithm proposed in this paper. Traditional features such as RMS and KI perform well in specific scenarios. However, when dealing with complex, non-stationary signals exhibiting impact-resonance modulation characteristics typical of bearing faults, a single feature is often insufficient. Bearing faults usually manifest as high-frequency resonance excited by periodic impacts, resulting in pronounced impulses and periodic structures in the envelope domain. To simultaneously capture both impulse sensitivity and periodic structure, the CFFI integrates FE and KI through normalized weighted fusion, defined as follows:

(1) $$CFFI\left(IMF_{i}\right)=w_{k}\times KInorm({IMF}_{i})+w_{e}\times [1-FEnorm({IMF}_{i})]$$

where $IMF_{i}$ represents the *i*-th intrinsic mode function; KInorm and FEnorm denote the normalized values of KI and FE for that IMF, respectively; $w_{k}$ and $w_{e}$ are weighting coefficients (default: $w_{k}$ = $w_{e}$ = 0.5, adjustable based on operational conditions). This definition ensures that a higher CFFI value indicates stronger impulses and periodic structures in the signal related to bearing faults. The CFFI serves both for selecting and clustering individual IMFs and as the fitness function for the Triangular Topology Aggregation Optimizer (TTAO) in optimizing VMD parameters, enabling the decomposition process to focus on fault-related features adaptively.

Fuzzy entropy [19] is used to quantify the complexity and uncertainty of a time series. Its value tends to be lower when the signal exhibits stronger periodicity. The calculation formula is as follows:

(2) $$\mathrm{FE}\mathrm{norm}\left(IMF_{i}\right)=-\ln\left(\frac{\phi_{m+1}\left(IMF_{i}\right)}{\phi_{m}\left(IMF_{i}\right)}\right)$$

where $\phi_{m}$ and $\phi_{m+1}$ represent the fuzzy similarity measures at embedding dimensions m and m+1, respectively.

The kurtosis index [20] quantifies impulse strength and the peakedness of a distribution. Impacts from bearing faults and the resulting pulses in the envelope domain typically cause a significant increase in kurtosis. It is calculated using the following formula:

(3) $$KInorm\left(IMF_{i}\right)=\frac{1}{N}\sum_{k=1}^{N}{\left(\frac{IMF_{i}\left[k\right]-\mu }{\sigma }\right)}^{4}$$

where *N* denotes the sample size, *μ* is the mean, and *σ* represents the standard deviation.

The construction described above ensures that the CFFI is simultaneously sensitive to both impulsive shocks and periodic structures. Consequently, a higher CFFI value indicates a more substantial presence of components associated with bearing faults. Therefore, the CFFI serves a dual purpose: it acts as a criterion for selecting relevant IMFs and functions as the fitness function for the TTAO optimization algorithm, guiding the adaptive search for optimal VMD parameters.

#### 2.1.2. Assessment Metric

To quantitatively evaluate the performance of the proposed novel health indicator (CFFI), this paper employs two standard metrics (Tred and Mono) along with one comprehensive metric, SAIF. Let the total number of sample segments be K, and the health indicator value for each segment be denoted as *h_k_*. The segment index *s_k_* can represent the time series. The definitions and significance of the three types of metrics are provided below.

(1) Tred: This metric measures the linear correlation between the health indicator and time, reflecting the overall change trend of the indicator as the fault evolves over time.
  (4) $$Tred=\frac{\left|\sum_{k=1}^{K}\left(h_{k}-\bar{H}\right)\left(s_{k}-\bar{S}\right)\right|}{\sqrt{\sum_{k=1}^{K}(h_{k}-\bar{H}{)}^{2}\sum_{k=1}^{K}(s_{k}-\bar{S}{)}^{2}}}$$
  where $\bar{H}$ is the mean value of the health indicator, and $\bar{S}$ is the average value of the time index. To enhance robustness, s_k_ can be assigned as the rank of the segment index.
(2) Mono: This metric measures the direction of change in the health indicator over time, which is crucial for evaluating the degradation process of equipment health. Monotonicity is defined as follows:
  (5) $$Mono=\frac{\left|N_{p}-N_{n}\right|}{K-1}$$
  where $N_{p}$ and $N_{n}$ represent the number of health indicators that are greater than 0 and less than 0 after differentiation, respectively, and K denotes the sample size.
(3) SAIF: This is used to comprehensively measure health indicators across multiple time scales, integrating methods such as multi-scale analysis, statistical features, and weighted summation. The definition of SAIF is as follows:
  (6) $$SAIF=\sqrt{\frac{1}{N}\sum_{i=1}^{N}w_{i}\left(\frac{1}{L}\sum_{j=1}^{L}{\left(\frac{\sigma_{ij}}{\mu_{ij}+\epsilon }\right)}^{2}+\alpha \cdot skew\left(x_{ij}\right)+\beta \cdot kurt\left(x_{ij}\right)\right)}$$
  where *N* is the number of time scales in the signal, *L* is the number of sub-windows within each time scale, $w_{i}$ is the weight of the ith time scale, $\sigma_{ij}$ and $\mu_{ij}$ are the standard deviation and mean of the *j*-th sub window under the *i*-th time scale, respectively, ϵ is a small constant to prevent division by zero, α and β are weight coefficients for skewness and kurtosis, and $skew(x_{ij})$ and $kurt(x_{ij})$ are the skewness and kurtosis of the *j*-th sub window under the *i*-th time scale, respectively.

### 2.2. Variational Mode Decomposition

VMD is an adaptive signal-processing method [21,22] that decomposes a complex signal into several IMFs with distinct center frequencies. It addresses issues such as mode mixing and end effects that are present in traditional EMD methods [23] and is widely applied in fields such as fault diagnosis and signal denoising. The core of VMD lies in minimizing the bandwidth of each mode through a variational approach. This process can be summarized in the following steps:

(1) Objective of Mode Decomposition:
  - The input signal *f(t)* is assumed to be decomposed into K mode functions *u_(k)_*, each associated with a center frequency *ω_k_*.
  - The objective is to find these mode functions *u_k_(t)* and their corresponding center frequencies *ω_k_* such that the bandwidth of each mode is minimized.
(2) Formulating the Variational Problem:

The problem of minimizing modal bandwidth is formalized as a constrained variational problem:

(7) $$\underset{\left\{u_{k}\right\},\left\{\omega_{k}\right\}}{min}\sum_{k=1}^{K}{∥\partial_{t}\left(\left(\delta \left(t\right)+\frac{j}{\pi t}\right)\;*\;u_{k}\left(t\right)\right)e^{-j\omega_{k}t}∥}_{2}^{2}\;s.t.\sum_{k}u_{k}=f$$

where $\partial_{t}$ denotes the time-derivative operator, $\delta \left(t\right)$ is the Dirac delta function, which ensures orthogonality of the modes in the time domain, ∗ represents the convolution operator, j is the imaginary unit, $u_{k}\left(t\right)$ is the kth mode function, and $\omega_{k}$ is its corresponding center frequency.

(3) Lagrangian Multiplier Method:

To handle the reconstruction constraint $\sum_{k=1}^{K}u_{k}\left(t\right)=f\left(t\right)$, the Lagrangian multiplier *λ(t)* is introduced:

(8) $$\begin{array}{c} L(\{u_{k}\},\{\omega_{k}\},\lambda )=\alpha \sum_{k=1}^{K}{∥\partial_{t}\left(\left(\delta \left(t\right)+\frac{j}{\pi t}\right)\;*\;u_{k}\left(t\right)\right)e^{-j\omega_{k}t}∥}_{2}^{2}+\\ {∥f\left(t\right)-\sum_{k=1}^{K}u_{k}\left(t\right)∥}_{2}^{2}+\left\langle \lambda \left(t\right),f\left(t\right)-\sum_{k=1}^{K}u_{k}\left(t\right)\right\rangle \end{array}$$

(4) Solving the Variational Problem: The Alternating Direction Method of Multipliers (ADMM) is employed to solve the aforementioned Lagrangian function iteratively, updating the mode functions $u_{k}(t)$ and the center frequencies $\omega_{k}.$
  (9) $$\begin{array}{cc} & u_{k}^{n+1}=\arg\underset{u_{k}}{min}{∥\partial_{t}\left(\left(\delta \left(t\right)+\frac{j}{\pi t}\right)\;*\;u_{k}\left(t\right)\right)e^{-j\omega_{k}t}∥}_{2}^{2}+\\ & \frac{\alpha }{2}{∥f\left(t\right)-\sum_{i\neq k}u_{i}\left(t\right)-u_{k}\left(t\right)+\frac{\lambda }{2\alpha }∥}_{2}^{2} \end{array}$$
  (10) $$\begin{array}{cc} & \omega_{k}^{n+1}=\frac{\int_{0}^{\infty }\omega |u_{k}(\omega ){|}^{2}d\omega }{\int_{0}^{\infty }|u_{k}(\omega ){|}^{2}d\omega }\\ & \lambda^{n+1}=\lambda^{n}+\tau \left(f\left(t\right)-\sum_{k=1}^{K}u_{k}^{n+1}\left(t\right)\right) \end{array}$$
(5) Convergence Criterion: It is determined whether the change in the mode functions $u_{k}$ during each iteration satisfies the convergence condition:
  (11) $$\sum_{k=1}^{K}\;\frac{∥u_{k}^{n+1}-u_{k}^{n}{∥}_{2}^{2}}{∥u_{k}^{n}{∥}_{2}^{2}}<\epsilon$$

If satisfied, the iteration stops. The motivation for selecting VMD as the modal decomposition method lies in its high degree of alignment with the mechanism of bearing faults: (1) It effectively captures and separates complex vibration modes. (2) It suppresses noise, thereby enhancing signal clarity. (3) It adapts to non-stationary signals, enabling precise analysis of transient features. (4) It identifies and distinguishes multiple fault modes.

### 2.3. Triangulation Topology Aggregation Optimizer

The Triangulation TTAO is a novel metaheuristic algorithm based on mathematical principles, specifically designed to address complex continuous optimization problems in engineering applications [24]. TTAO leverages the geometric properties of similar triangles to balance exploration and exploitation, thereby improving the efficiency of finding the optimal solution.

The core concept of TTAO is to partition the search space into multiple triangular topology units. Each unit is optimized by updating its vertices and generating interior points within the triangle. These units iteratively evolve through processes of global and local aggregation, gradually converging towards the optimal solution.

The TTAO algorithm is implemented through the following steps:

(1) Initialization: An initial population P is distributed within the search space, forming several triangular units. Each unit consists of three vertices $X_{i1}$, $X_{i2}$, $X_{i3}$, and an additional point $X_{i4}$
  (12) $$P=\left\{X_{1},X_{2},\ldots ,X_{N}\right\}$$
  where N represents the population size, and each $X_{i}$ is a d-dimensional vector.
(2) Formation of Triangular Units: The vertices $X_{i1}$, $X_{i2}$, and $X_{i3}$ of each triangular unit can be randomly distributed within the search space during the initialization process. The additional point $X_{i4}$ is typically generated inside the triangle to assist in the optimization process.
(3) Global Aggregation: During the Global Aggregation phase, new candidate solutions are generated by performing weighted combinations of the vertices from different triangular units. This step emphasizes exploring potential regions within the search space.

Assuming the vertices of a triangular unit $\Delta_{ijk}$ are $X_{i}$, $X_{j}$, and $X_{k}$, a new point $X_{new}$ can be generated using the following formula:

(13) $$X_{new}=\alpha X_{i}+\beta X_{j}+\gamma X_{k}$$

where *α*, *β*, *γ* are weighting coefficients that satisfy *α* + *β* + *γ* = 1, and are typically determined using random methods.

(4) Local Aggregation: The Local Aggregation phase focuses on refining the search within each triangular unit. A local search around the best vertex is conducted by perturbing its position to find a better solution. For each vertex $X_{i}$, its update can be expressed as:
  (14) $$X_{i}^{\left(t+1\right)}=X_{i}^{\left(t\right)}+\lambda \cdot \left(X_{best}-X_{i}^{\left(t\right)}\right)$$
  where *λ* is a step-size coefficient, $X_{\mathrm{best}}$ is the best-performing vertex in the current triangular unit, and t denotes the iteration number.
(5) Iteration and Convergence: The algorithm alternates between global and local aggregation phases, updating the individuals in the population until a convergence condition is met (e.g., a maximum number of iterations or an acceptable solution quality). The convergence criterion can typically be defined as the change in the objective function value Δf<ϵ, or reaching a predefined maximum number of iterations $t_{\max}$: $\Delta f=|f(X^{\left(t\right)})-f(X^{\left(t-1\right)})|<\epsilon$ or $t\geq t_{max}.$

### 2.4. Dynamic Weighted Centroid Clustering

In signal analysis, decomposing a complex signal into several IMFs with distinct characteristics is a crucial step. Building on this, clustering the IMFs can further reveal underlying patterns and structures within the signal. To better analyze and utilize the decomposed IMFs, this section proposes a DWCC method, which is described in detail alongside actual vibration signals. The specific steps of the DWCC algorithm are as follows:

(1) Data Standardization: Prior to cluster analysis of the IMFs, it is necessary to standardize the CFFI values of the IMFs. This process aims to eliminate the influence of IMFs with different magnitudes and scales on the clustering results, allowing for comparison and analysis of all IMFs on a unified scale. The standardized CFFI value is defined as
  (15) $${CFFI}_{normalized}\left(IMF_{i}\right)=\frac{CFFI\left(IMF_{i}\right)-\mu_{CFFI}}{\sigma_{CFFI}}$$
  where ${\mathrm{CFFI}}_{normalized}({\mathrm{IMF}}_{i})$ is the CFFI value of the *i*-th IMF. $\mu_{\mathrm{CFFI}}$ and $\sigma_{\mathrm{CFFI}}$ are the mean and standard deviation of the CFFI values for all IMFs, respectively.

Through standardization, the impact of differences in magnitude across IMFs is eliminated, thereby improving the accuracy and effectiveness of subsequent cluster analysis.

(2) Cluster Center Initialization: After standardization, the K-means++ algorithm is employed to select K initial cluster centers $\left\{\mu_{1},\mu_{2},\cdots ,\mu_{K}\right\}$, which are determined using the following formula:
  (16) $$\mu_{j}^{\left(0\right)}=\arg\underset{i}{min}∥{\mathrm{CFFI}}_{\mathrm{normalized}}\left(IMF_{i}\right)-\mu_{j}{∥}^{2}$$
  where $\mu_{j}^{\left(0\right)}$ denotes the initial center of the *j*-th cluster. The K-means++ algorithm ensures a reasonable distribution of the initial centers, thereby improving the convergence speed and the effectiveness of the clustering results.

(3) Improved Distance Metric: In DWCC, an improved weighted Euclidean distance metric is used to calculate the similarity between each IMF and the cluster centers, enabling precise measurement of the similarities among IMFs:
  (17) $$d_{w}\left(IMF_{i},\mu_{j}\right)=\sqrt{\sum_{k=1}^{m}w_{k}{\left({CFFI}_{\mathrm{normalized},k}\left(IMF_{i}\right)-\mu_{j,k}\right)}^{2}}$$
  where $w_{k}$ is the weight for each feature. m is the dimensionality of the features.
(4) Adaptive IMF Assignment: An adaptive distribution strategy is employed, which dynamically assigns IMFs to the most suitable cluster center based on the local density and global similarity of each IMF. The adaptive IMF assignment strategy is defined as
  (18) $$c_{i}=\arg\underset{j}{min}\left(d_{w}\left(IMF_{i},\mu_{j}\right)+\lambda \frac{1}{\rho \left(IMF_{i}\right)}\right)$$
  where $c_{i}$ is the assigned class of ${\mathrm{IMF}}_{i}$. λ is a balancing factor used to adjust the influence of distance and density. $\rho ({\mathrm{IMF}}_{i})$ represents the local density of ${\mathrm{IMF}}_{i}$.
(5) Dynamic Cluster Center Update: Cluster centers are updated dynamically using a weighted averaging method to achieve smooth transitions and accelerate convergence. The dynamic update strategy is as follows:
  (19) $$\mu_{j}^{\left(t+1\right)}=\alpha \mu_{j}^{\left(t\right)}+\left(1-\alpha \right)\frac{1}{\left|C_{j}\right|}\sum_{i\in C_{j}}{CFFI}_{\mathrm{normalized}}\left(IMF_{i}\right)$$
  where α is a smoothing coefficient that controls the weight distribution between the new cluster center and the old one. $C_{j}$ is the set of IMFs currently belonging to the jth cluster. $|C_{j}|$ is the number of IMFs in the jth cluster.
(6) Adaptive Convergence Criterion: The convergence criterion is dynamically adjusted based on the stability and trend of the cluster centers to ensure the algorithm achieves optimal clustering results within a reasonable timeframe:
  (20) $$∥\mu_{j}^{\left(t+1\right)}-\mu_{j}^{\left(t\right)}∥<\epsilon_{t}\forall j$$
  where $\epsilon_{t}$ is the convergence threshold at the t-th iteration, which can be dynamically adjusted according to the historical changes in the cluster centers.
(7) Optimization Enhancement Strategy: After each iteration, a local search and optimization are performed on the assignment results to reduce intra-class variance further and enhance the robustness and accuracy of the clustering. Specific measures include: reassigning and optimizing IMFs within each cluster, removing IMFs that may belong to multiple categories, and adjusting them to more suitable classes.

### 2.5. Algorithm Flow

In the method proposed in this paper, the novel health indicator CFFI is used as the fitness function given in Equation (21), and TTAO is employed to optimize VMD. (* indicates specific numerical values used in practical operation.)

(21) $$\left\{\begin{array}{c} fitness=\underset{\left(K,\alpha \right)}{min}\left({CFFI}_{i}\right)\\ s.t.K\in \left(*,*\right)\\ \alpha \in \left[*,*\right] \end{array}\right.$$

**Figure 1:**
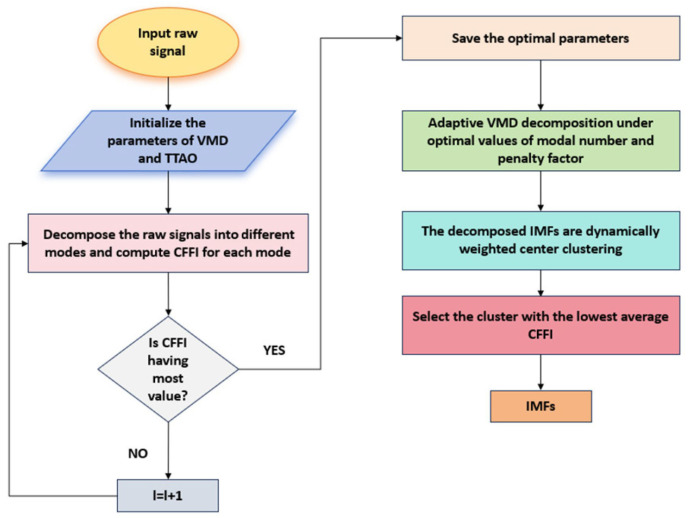
Algorithm flowchart.

${CFFI}_{i}$ is the fitness function value obtained for each candidate. The proposed method is illustrated in Figure 1.

1. Initialize the parameters of TTAO and VMD.
2. Decompose the original signal into different modes.
3. Calculate the CFFI for each modality.
4. Calculate the fitness function, CFFI, for each modality until the maximum iteration is reached.
5. Obtain the global optimal fitness function and save the optimal values of the mode number K and the penalty factor α.
6. Perform DWCC on the decomposed IMFs.
7. Select the cluster with the highest average CFFI value as the IMFs for the reconstructed signal.

In practical decomposition, low-energy narrowband residual or broadband noise IMF usually exhibits lower CFFI values. DWCC will classify them into low CFFI clusters and remove them in the reconstruction stage, thereby improving the energy concentration and identifiability of fault-related components.

## 3. Simulation Analysis

**Figure 2:**
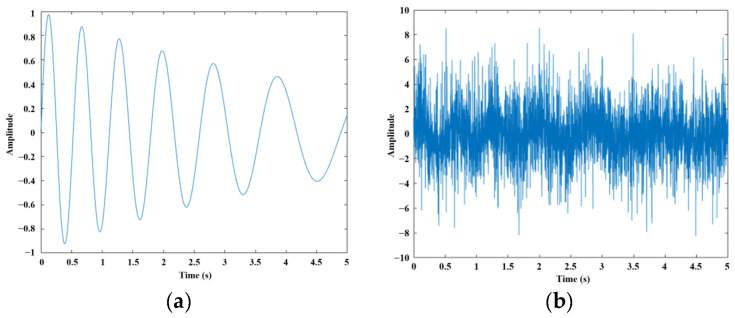
(**a**) Pure periodic decreasing signal; (**b**) periodic decreasing signal perturbed by white noise.

To validate the applicability of the proposed method during bearing operation, a non-stationary simulated signal was constructed as a benchmark. This signal is designed to evaluate the algorithm’s capabilities for decomposition, optimization, and reconstruction under time-varying frequency and amplitude conditions, as well as in the presence of background noise. It is important to note that this simulated signal is not a physical model of a bearing fault but rather a non-stationary test sequence used to assess the algorithm’s robustness. The base signal is defined as a sinusoidal wave whose period and amplitude decrease over time via time attenuation factors. The mathematical expressions for a periodic signal with period T are given as:

(22) $$f\left(t\right)=f_{0}\cdot e^{-k_{f}t}$$

(23) $$A\left(t\right)=A_{0}\cdot e^{-k_{A}t}$$

(24) $$x_{sin}\left(t\right)=A\left(t\right)\cdot \sin\left(2\pi f\left(t\right)t\right)$$

where A is the initial amplitude of the signal, f is the signal frequency, t is time, $f_{0}=2\;\mathrm{Hz}$, $k_{f}=0.1\;s^{-1}$, $A_{0}=1$, and $k_{A}=0.2\;s^{-1}$, as shown in Figure 2a.

On this basis, random white noise is generated and added to the above signal to form the final simulated signal, as shown in Figure 2b, expressed as:

(25) $$x_{final}\left(t\right)=x_{sin}\left(t\right)+n\left(t\right)$$

where $n(t)=(a_{min}+(a_{max}-a_{min})\cdot r)\cdot N(0,1)$, with $a_{min}=0.1$, $a_{max}=3.0$, r being a random number uniformly distributed in [0, 1], and N(0,1) representing Gaussian noise with zero mean and unit variance.

**Figure 3:**
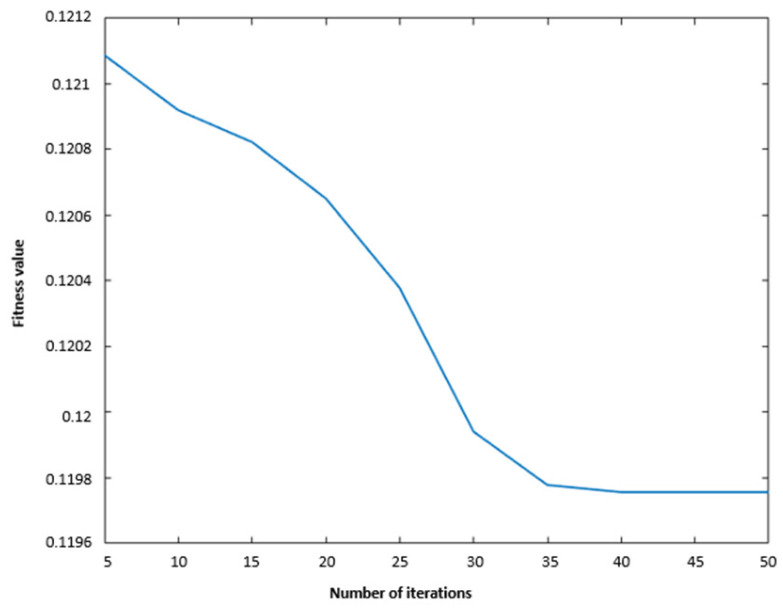
Iterative curve.

**Figure 4:**
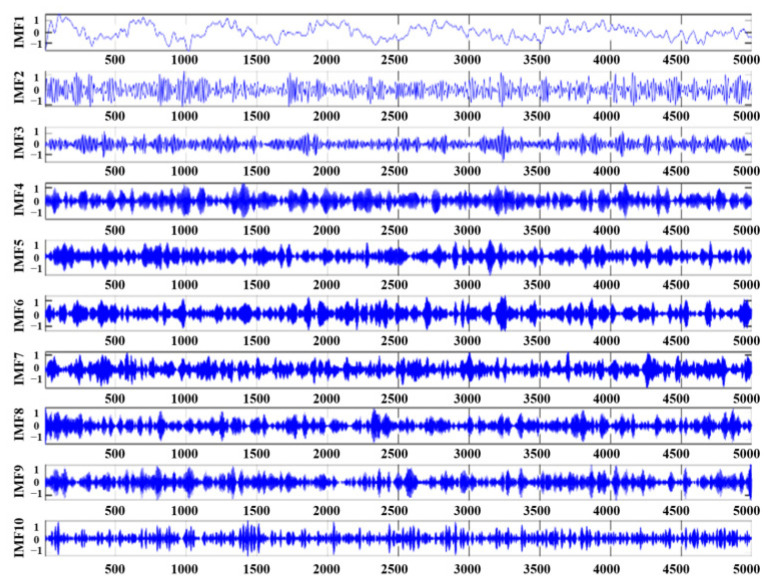
The IMFs after decomposition.

**Figure 5:**
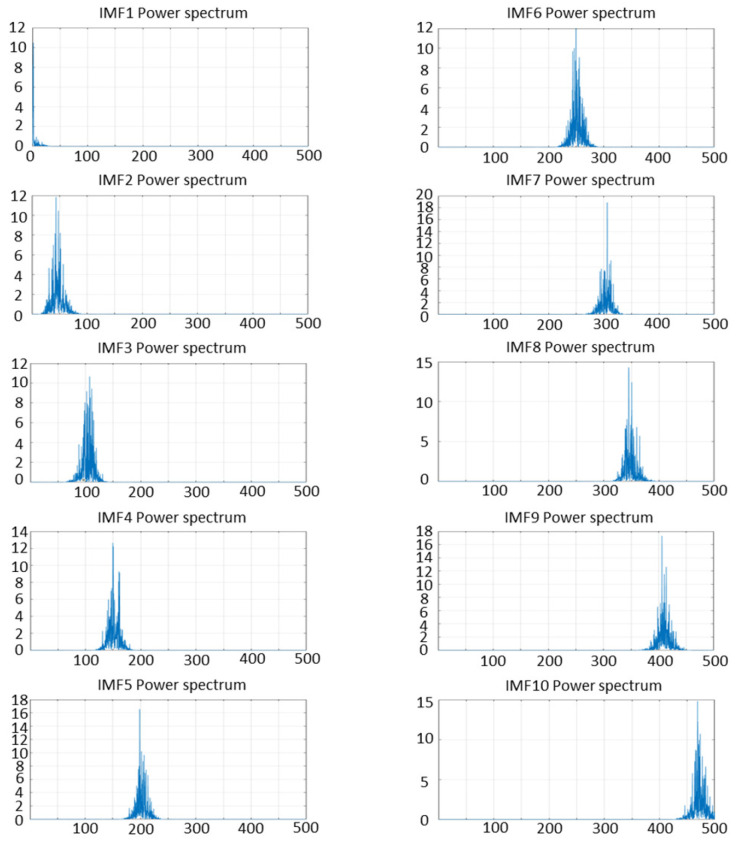
Frequency corresponding to IMFs.

First, the parameters for VMD and TTAO were initialized, with CFFI set as the fitness function for TTAO, aiming to maximize fitness. For the VMD, the number of iterations was set to 100, and the maximum number of decomposition layers $K_{\max}=25$. For the TTAO optimization, the number of iterations was set to 50. The resulting optimal fitness value found by TTAO was 0.11976 (as shown in Figure 3). Subsequently, VMD was used to decompose the original signal into several IMFs with different center frequencies. During each decomposition and parameter search, the CFFI was calculated for each IMF and aggregated (e.g., by taking the maximum value or the mean of the top p values) to form the fitness value. This process iterated until the parameter combination yielding the highest fitness was found, and the optimal parameters were saved (the optimal number of modes K=10, and the penalty factor α=200). Under these parameters, the corresponding IMFs (see Figure 4) and their center frequencies (see Figure 5) were obtained.

**Figure 6:**
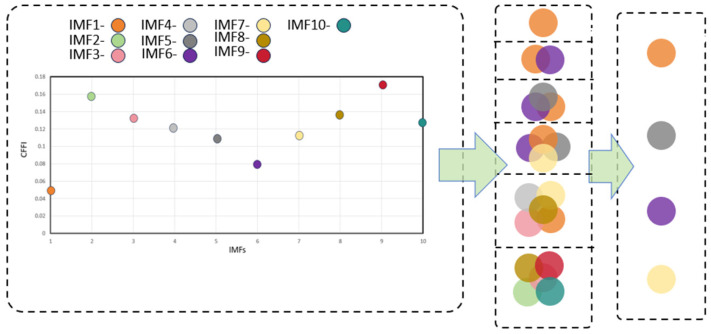
CFFI and DWCC select IMFs.

As shown in Figure 4 and Figure 5, the first three IMFs clearly capture the pulse-resonance-dominated shock-attenuation characteristics; the subsequent IMF (IMF4-IMF10) mainly includes low-energy narrowband residual and broadband noise preserved after optimized parameter decomposition (K = 10). Based on the screening of CFFI and DWCC, these low-CFFI modes are classified as noise clusters and discarded in the reconstruction, further ensuring the feature set and denoising effect of the reconstructed signal. DWCC was then performed on all IMFs, followed by CFFI-based screening. The cluster with the highest average CFFI was selected for reconstruction to focus on fault-related components (four components were obtained, as shown in Figure 6).

In this example, the number of clusters was set to six. Since a higher CFFI value indicates stronger fault-related impulses and periodic structures, the cluster with the highest average CFFI (in this case, comprising four IMFs) was selected to form the reconstruction components. To evaluate denoising and feature-preservation performance, the SERR is introduced. Without requiring a clean reference signal, SERR assesses the overall quality of noise reduction and feature extraction by comparing the energy changes in the frequency domain before and after denoising. It is defined as

(26) $$E_{original}=\sum_{k}|Y_{original}\left[k\right]{|}^{2}$$

(27) $$E_{denoised}=\sum_{k}|Y_{denoised}\left[k\right]{|}^{2}$$

(28) $$SERR=\frac{E_{denoised}}{E_{original}}$$

where $Y_{\mathrm{original}}[k]$ is the spectral representation of the signal before denoising, $Y_{\mathrm{denoised}}[k]$ is the spectral representation of the signal after denoising, $E_{\mathrm{original}}$ represents the total spectral energy of the signal before denoising, and $E_{\mathrm{denoised}}$ represents the total spectral energy of the signal after denoising.

**Figure 7:**
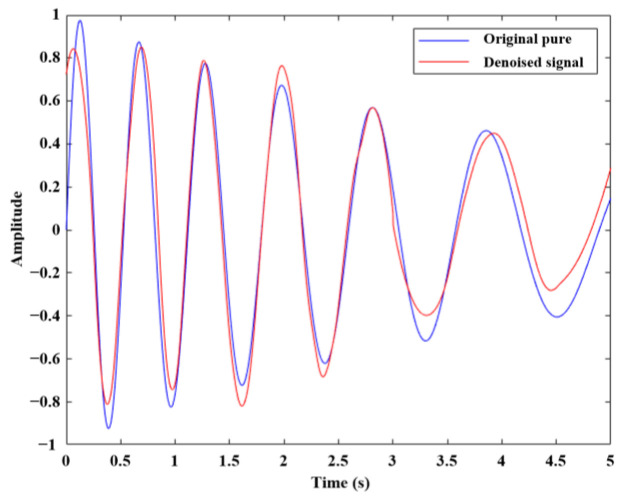
Contrast diagram of pure signal and denoised signal.

**Figure 8:**
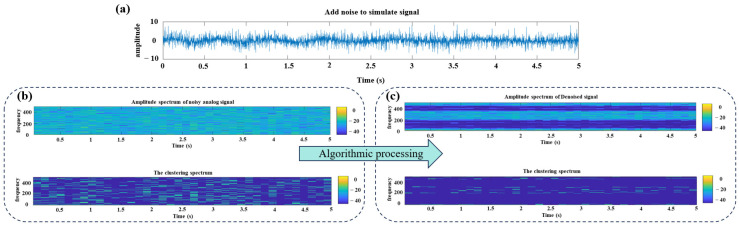
Analog signal spectrum energy change. (**a**) time-domain waveform containing noisy signals; (**b**) amplitude spectrum and clustering spectrum of the noise signal; (**c**) amplitude spectrum and clustering spectrum of the denoised signal.

Finally, the four obtained components were reconstructed (as shown in Figure 7, which compares the reconstructed signal with the pure signal, demonstrating effective denoising). The spectral energy of the reconstructed signal was calculated, resulting in an SERR value of 0.31254. A lower SERR value indicates a greater reduction in the denoised signal’s overall spectral energy, indicating good denoising performance. Figure 8 better illustrates the specific implementation of the signal processing. Figure 8a shows the time-domain plot of the generated noisy signal, and Figure 8b displays the amplitude spectrum and cluster spectrum of the noisy signal. Under the influence of noise, the signal’s spectrum appears complex and difficult to analyze directly. Figure 8c presents the amplitude spectrum and cluster spectrum of the denoised signal after processing by the proposed algorithm. Using this algorithm, noise is effectively removed, significantly enhancing signal clarity and feature extraction accuracy.

As shown in Figure 8, the processed signal exhibits more concentrated spectral energy and reduced noise, validating the algorithm’s effectiveness for signal denoising and feature extraction.

To verify the algorithm’s stability, 10 additional simulation trials were conducted. The parameters for the simulated signals are listed in Table 1. Results from multiple trials demonstrate that the proposed method maintains good consistency and robustness under non-stationary and noisy conditions.

In summary, the signal-processing framework proposed in this paper applies to the analysis of non-stationary signals with decaying periods. It demonstrates good adaptability for feature extraction in bearing vibration signals.

## 4. Experimental Validation

To verify the practical application advantages of CFFI in bearing fault detection, comparative experiments were conducted based on the IMS public dataset. The overall process includes data preprocessing, indicator calculation and evaluation, decomposition and optimization, IMF screening and reconstruction, as well as ablation and repeated experiment analysis.

Data and Preprocessing:

(1) Frequency band selection and envelope extraction: The failure of the bearing body is usually manifested as periodic impact excitation of high-frequency resonance, and the formation of low-frequency amplitude modulation characteristics in the envelope domain. Combine known or estimated characteristic frequencies, or select bands based on spectral kurtosis to determine the bandpass range, using zero-phase bandpass filtering to preserve fault-related frequency bands; subsequently, Hilbert transform is applied to the bandpass signal to obtain the envelope, and slight detrending is performed as needed to eliminate DC bias. This process can suppress irrelevant noise while highlighting amplitude modulation and pulse information.
(2) Standardization: Standardize the envelope or bandpass signal with z-scores to achieve zero mean and unit variance, improving comparability across different operating conditions/time periods.
(3) Segmented processing: Segment the standardized signal into fixed time windows (approximately 20,480 points per window), and calculate CFFI, RMS, KI, and evaluation metrics (Trend, Monotonity, SAIF) window-by-window to maintain temporal resolution and characterize dynamic changes.

**Figure 9:**
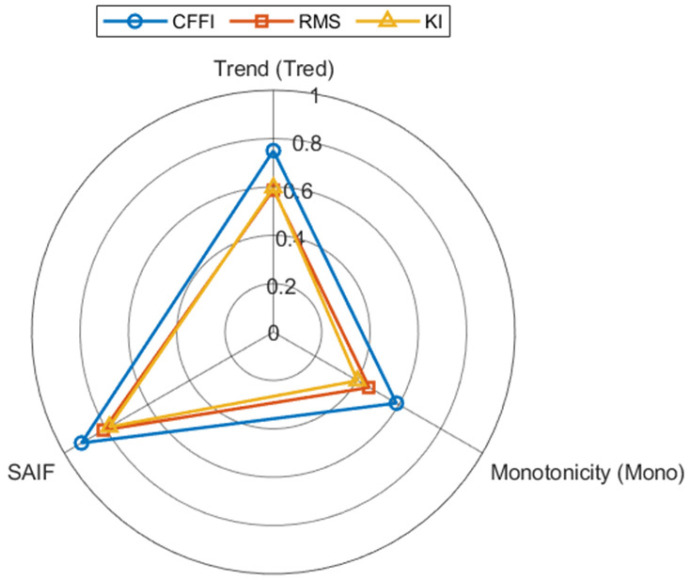
Radar chart comparison of the health indicators across Tred, Mono, and SAIF.

CFFI, root mean square (RMS), and kurtosis index (KI) were calculated window-by-window on IMS data, and evaluated and compared using three metrics: trend, monotonicity, and scale-adaptive integrated feature variability (SAIF). The summary results are shown in Figure 9.

The results showed that CFFI had advantages in all three metrics, especially in the SAIF dimension approaching 1, indicating its stronger significance and stability in fault-related features at multiple scales. Combining the composite characterization of pulse-like (KInorm) and periodic structure complexity (1-FEnorm) in CFFI, it can be concluded that its advantage comes from simultaneously characterizing peak and periodic structures, thereby more robustly highlighting fault-related features under different operating conditions and time scales. This further validates the sensitivity and robustness of CFFI as a health assessment indicator for bearings.

The sampling rate for IMS data recording is 20 kHz, with approximately 20,480 points per file. The preprocessing stage determines the fault-related resonance band based on spectral kurtosis and its harmonics, and performs envelope demodulation and trend removal. Subsequently, the envelope domain signal is decomposed into IMFs with different center frequencies using VMD, with a fixed number of iterations of 100 and a maximum decomposition level of K_max_ = 25. To obtain the optimal decomposition configuration, TTAO is introduced to adaptively search for the penalty factor α and mode number K, and the optimization budget is uniformly set to 50 evaluations; in each candidate parameter evaluation, calculate the CFFI of each IMF and update the search parameters with the goal of maximizing CFFI. The representative results show that the optimal parameters are α = 400 and K = 10. Under this configuration, the SERR of the reconstructed signal is 0.21356, which is lower than the value of 0.22465 for the actual collected signal. This reduction in SERR indicates a decrease in extraneous spectral energy, thereby confirming the effectiveness of the denoising process and a higher quality of decomposition and reconstruction. The relative error between the two is 4.9%. Subsequently, the CFFI mean of each cluster is calculated, and the cluster with the highest mean is selected as the reconstruction set to avoid irrelevant or redundant modes from being mixed in. This process usually selects representative components such as IMF1, IMF5, IMF10 for reconstruction in experiments, which not only preserves the peak and amplitude modulation structure of fault pulses, but also effectively suppresses background noise and non-fault components.

**Figure 10:**
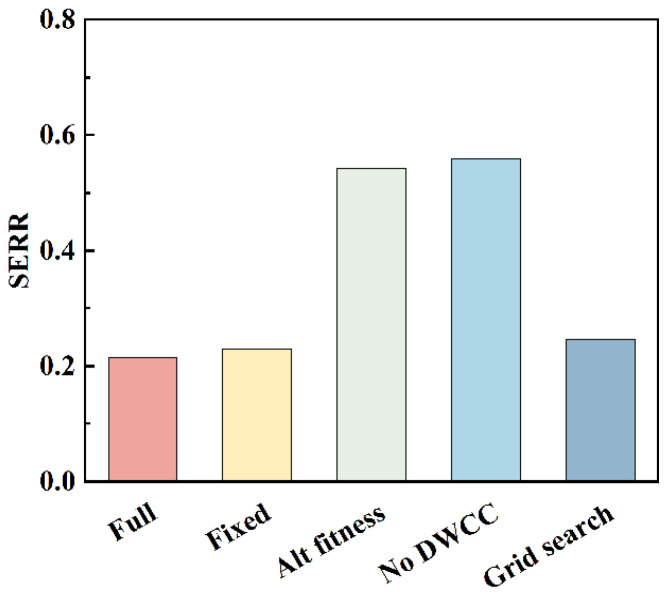
Ablation experiment analysis.

On the premise of ensuring consistency in the data, preprocessing, VMD iteration, and optimization budget, ablation experiments were conducted to quantify the independent contributions of each module. Figure 10 compares the overall performance of five methods on SERR: (1) the complete method (TTAO+CFFI+DWCC); (2) the fixed parameters (K = 10, α = 400) are slightly higher than the complete method, indicating that empirical parameters can approach the optimum under specific operating conditions but lack adaptability; (3) under the same budget, the SERR of the grid search further increases, reflecting the influence of step size and discretization constraints; (4) replacing CFFI with minimum envelope entropy as the fitness resulted in a significant increase in SERR, indicating that entropy indices are less sensitive to spike pulses and complex changes than CFFI; (5) excluding DWCC and directly selecting the top K IMFs for reconstruction results in mixed information and energy dispersion significantly compromised the quality of reconstruction and diagnostic distinguishability.

**Figure 11:**
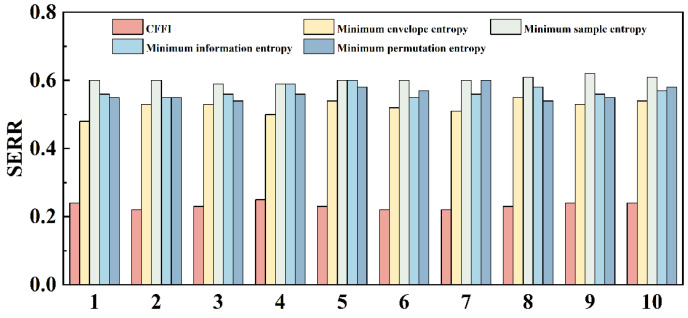
Comparative analysis of SERR under different fitness functions.

In addition, Figure 11 compares the SERR performance of different fitness functions in 10 repetitions. When using CFFI as the fitness, the 10 results remained stable in the range of approximately 0.20–0.24 with minimal fluctuations; when using minimum envelope entropy, minimum information entropy, minimum sample entropy, and minimum permutation entropy, the overall SERR significantly increases (typical range of about 0.50–0.60), and the dispersion across experiments significantly increases. This result indicates that CFFI can more directly measure the peak characteristics and complex dynamic changes of fault pulses, providing a clear convergence direction for optimizers; entropy-based indicators are susceptible to distribution estimation and window settings in noisy backgrounds and non-stationary pulse-dominated scenes, leading to unstable parameter selection and decreased reconstruction quality. Based on the data from different comparisons and repeated experiments, it can be concluded that TTAO is more efficient in approaching optimal solutions than grid search under limited evaluation budget; CFFI, as a fitness function, is more suitable for characterizing the pulse peaks of mechanical faults than entropy-based indicators; DWCC is a key step in ensuring the quality of IMF screening, which can improve the energy concentration of reconstruction and the significance of envelope spectral fault features.

## 5. Conclusions

A health indicator-driven IMF clustering method for bearing fault detection was proposed. To address common issues in non-stationary and noisy environments, such as the susceptibility to mode mixing in traditional methods, difficulty in parameter self-adaptation, and lack of a closed-loop assessment, a framework for adaptive decomposition and selection guided by a CFFI was constructed. Using CFFI as both a sensitive and robust health indicator and an optimization objective, the method employs a TTAO to adaptively search for the optimal penalty factor α and mode number K for VMD. This enabled the decomposition to focus on fault-related impulse and amplitude-modulated resonance modes. Subsequently, DWCC was applied to screen the obtained IMFs, and the cluster with the highest average CFFI value—based on the semantic principle that “higher CFFI indicates stronger fault-related components”—was selected for reconstruction. Finally, a closed-loop assessment is based on the SERR, where the global full-spectrum SERR was used to measure overall noise reduction, and the feature-band SERR measures retention of fault characteristics. The experimental and simulation results based on the IMS public bearing dataset show that the proposed method achieved stable parameter estimation and clear fault modes under various operating conditions. Compared to baseline schemes using RMS, KI, or various entropy-based fitness functions, the proposed method exhibited higher Tred/Mono and SAIF. It achieved a significant reduction in the global SERR while maintaining a stable high retention in the feature-band SERR. This reflected its stronger sensitivity, robustness, and online applicability. It was noted that the current validation primarily relied on the IMS dataset; thus, the method’s generalizability across different bearing types and more-variable operational conditions is suggested to warrant further investigation. Future work will focus on automating frequency band selection and CFFI weight adaptation, validating generalization under complex variable-speed conditions, and enabling real-time deployment with computational optimization for embedded platforms.

## Figures and Tables

**Table 1:** Calculation of analog signal SERR.

Number	SERR
1	0.31526
2	0.41265
3	0.34512
4	0.31569
5	0.36965
6	0.37445
7	0.40168
8	0.35487
9	0.30875
10	0.32588

## Data Availability

The data are contained within the article.

## Abbreviations

The following abbreviations are used in this manuscript:

SAIF	Scale-Adaptive Integrated Feature Variability
TTAO	Triangular Topology Aggregation Optimizer
DWCC	Dynamic Weighted Central Clustering
CFFI	Composite Fault Feature Indicator
VMD	Variational Mode Decomposition
SERR	Spectral Energy Retention Rate
IMFs	Intrinsic Mode Functions
SNR	Signal-to-Noise Ratio
RMS	Root Mean Square
Mono	Monotonicity
FE	Fuzzy Entropy
KI	Kurtosis
Tred	Trend
